# Supplementary figures and images for: Genome-wide analysis, identification, evolution and genomic organization of dehydration responsive element-binding (DREB) gene family in Solanum tuberosum
Source: PeerJ. 2021 Jun 24;9:e11647. doi: 10.7717/peerj.11647 (PMC8236231; doi:10.7717/peerj.11647)

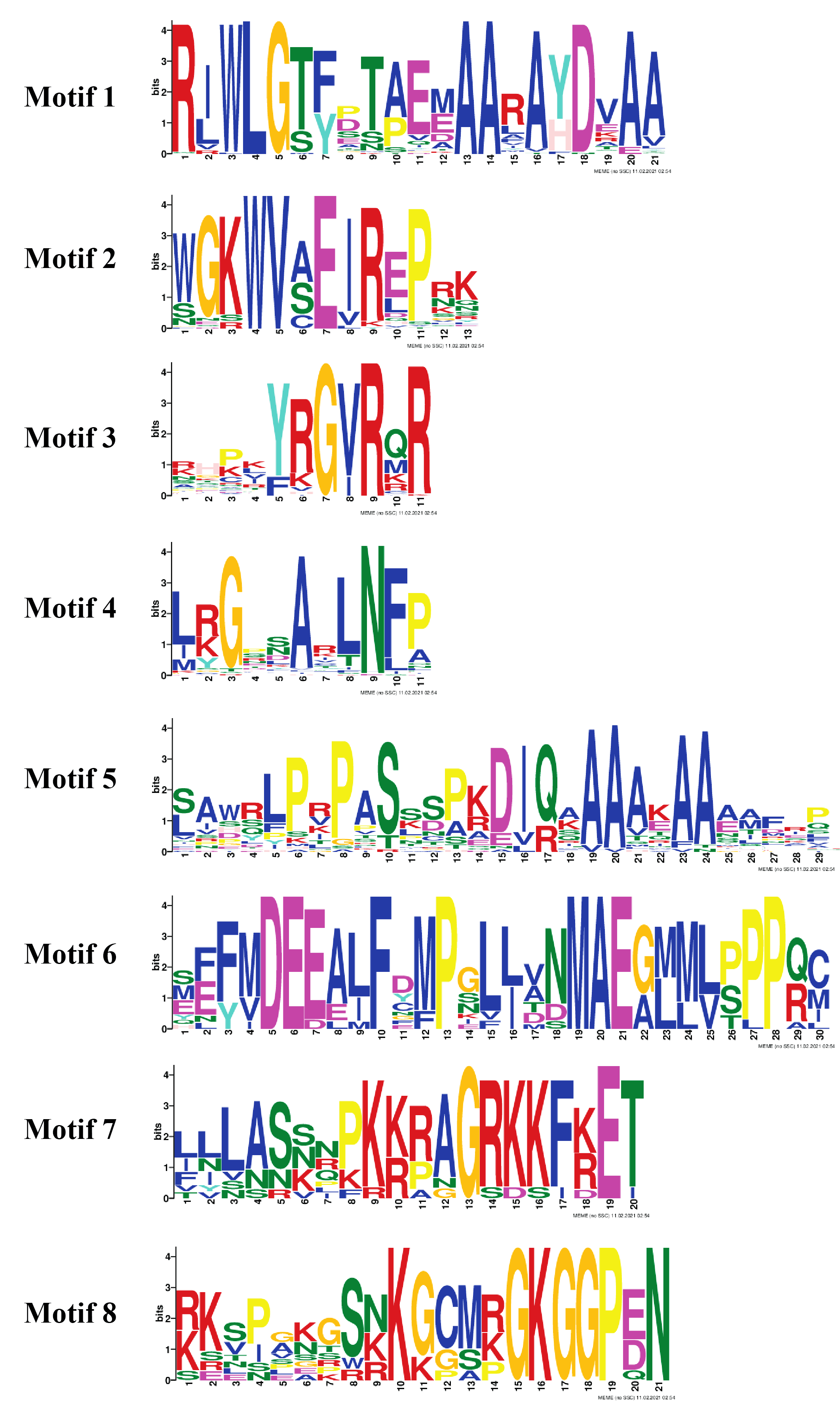

Supplement: Supplemental Information 10 [file peerj-09-11647-s010.png]

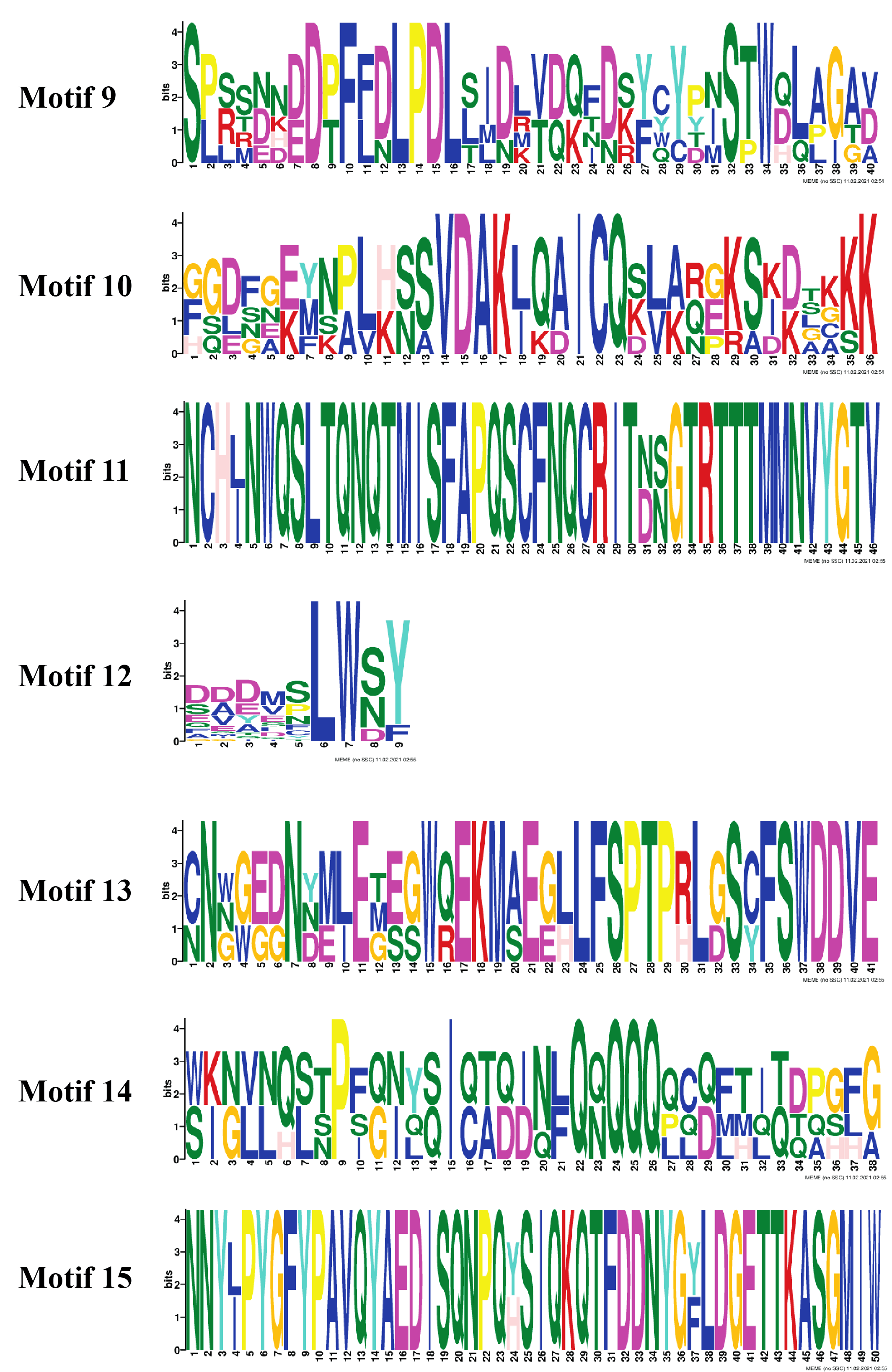

Supplement: Supplemental Information 11 [file peerj-09-11647-s011.png]
